# Protective Effect of N-Acetyl Cysteine on Chlorpyrifos-Induced Testicular Toxicity in Mice

**DOI:** 10.22074/ijfs.2019.5494

**Published:** 2019-01-06

**Authors:** Rasoul Kheradmandi, Seyed Gholam Ali Jorsaraei, Farideh Feizi, Ali Akbar Moghadamnia, Nahid Neamati

**Affiliations:** 1 Department of Anatomical Sciences, Babol University of Medical Sciences, Babol, Iran; 2 Fatemeh-Zahra Infertility and Health Reproductive Research Center, Babol University of Medical Sciences, Babol, Iran; 3 Department of Pharmacology, Babol University of Medical Sciences, Babol, Iran; 4 Department of Clinical Biochemistry, Babol University of Medical Science, Babol, lran

**Keywords:** Chlorpyrifos, N-acetylcysteine, Protective, Sperm

## Abstract

**Background:**

Chlorpyrifos (CPF), an organophosphate pesticide, is widely used in farms in order to preserve crops and fruits. Previous studies have shown that CPF exposure might cause chronic toxicity in male genital system. The present study investigated the protective effect of N-Acetyl Cysteine (NAC), a potent antioxidant against testicular toxicity of CPF in male mice.

**Materials and Methods:**

In this experimental study, 42 adult male mice were divided into seven groups, CPF low (0.5 mg/kg.b.w) and high (5 mg/kg.b.w) doses groups, NAC group (35 mg/kg.b.w), NAC+CPF 0/5 mg/kg.b.w, NAC+CPF 5 mg/kg.b.w, dimethyl sulfoxide (DMSO, 0.75% solution mg/kg.b.w) and control group. All treatment were done intraperitoneally. Treatment was conducted for four consecutive weeks (five days each week). However NAC was injected to NAC+CPF groups five days before initiation of the treatment procedure. One week after the last injection, mice were sacrificed using anesthetic gas to evaluate alterations in testicular histology and sperm parameters.

**Results:**

Seminiferous tubules area and diameter were significantly diminished in the group treated with 5 mg/kg CPF (P<0.05). CPF also statistically reduced sperm parameters (count and motility) and damaged sperm morphology) at both doses (P<0.05). However, NAC significantly improved spermatogonia, spermatocytes, spermatid cell counts as well as sperm parameters in mice treated with both CPF concentrations (P<0.05).

**Conclusion:**

According to our results, NAC may significantly ameliorate CPF-induced damages to spermatogonia, spermatocytes, spermatids cell counts and sperm parameters.

## Introduction

As reported by the World Health Organization (WHO), unsuccessful pregnancy has been globally increased. Researchers found that 48.5 million couples worldwide were unable to have a child after five years of unprotected regular sexual intercourse (1).

Almost all people working on agricultural fields are exposed to various toxins that may cause reproductive toxicity. Pesticides are widely used for eliminating pests to protect corps and fruits. Organophosphate pesticides are regarded as dangerous types of pesticides for the environment as they can affect humans and animals health (2). Chlorpyrifos (O,O-Diethyl O-3,^5^,^6^-trichloropyridin- 2-yl phosphorothioate), is an organophosphate pesticide which can cause adverse effects on the reproductive system of both males and females (3). For instance, seminiferous tubules were significantly degenerated in chlorpyrifos (CPF)-treated mice (4). In addition, sexual hormones disturbance and also defects in sperm production have been reported following CPF exposure (5). CPF was also shown to increase DNA impairment (6, 7) and induce harmful effects in different organs such as the thyroid (8) and lung (9). CPF permanently binds acetylcholinesterase and inhibits deactivation of acetylcholine in the synapses. So, acetylcholine signaling may last longer. This process is irreversible unless new acetylcholinesterase enzymes are synthesized. It has been reported that CPF also induces oxidative stress (8, 10).

Acetylcysteine, also known as N-acetyl cysteine (NAC) is widely used in management of acetaminophen overdose, cystic fibrosis and also chronic obstructive pulmonary disease. NAC may be useful in toxins treatments, since it can escalate glutathione levels and prevent further injuries caused by lipid peroxidation (11). Furthermore, a significant improvement of sperm motility and morphology were observed by NAC treatment in varicocele and also other models induced by synthetic drugs such as paracetamol (12, 13). Moreover, NAC may protect male genital system against strong toxins such as arsenic trioxide in (14). It has been observed that NAC has more marked effects compared to vitamin C in improvement of sperm parameters (15). Therefore, this study was conducted to investigate the protective effect of NAC on histopathology of testis and sperm parameters in CPF-treated mice. 

## Materials and Methods

### Chemicals

Chlorpyrifos (99%) and NAC (99%) technical grade were purchased from Sigma-Aldrich (St. Louis, MO, USA) [lot No. LC13116V and 616-91-1, respectively]. Also dimethyl sulfoxide (DMSO) was provided from Sigma too (St. Louis, MO, USA) [Lot No. 67-68-5].

### Experimental design

Forty-two healthy adult BALB/c mice (6-8 weeks old) were obtained from the Animal Research Unit, Babol Medical University, Babol. Animal care and handling was done based on Animal Research Unit and following approval of Ethics Committee (MUBABOL.HRI. REC.1395.73). The animals were habituated to laboratory conditions for 1 week before initiation of the experiment. Mice were maintained on 12 hours light-dark cycle at 21-24°C with 50-60% humidity. Mice had free access to normal diet and water, ad libitum. The animals were divided into seven groups: group I (control group) received normal saline, group II (sham group) received DMSO (0.75% solution), group III received NAC 35 mg/kg.b.w, group IV (high CPF) received CPF 5 mg/kg. b.w, group V (low CPF) received CPF 0.5 mg/kg.b.w, and group VI and VII received CPF at low (0.5 mg/kg.b.w) and high (5 mg/ kg.b.w) doses, respectively along with NAC on a daily basis. In groups VI and VII, NAC was given intraperitoneally from five days before the experimental timeline, in order to acclimate mice with this antioxidant. All groups were treated intraperitoneally except the control group. Treatment was conducted for 4 weeks and injections in all groups were administrated on five consecutive days each week. One week after the last injection, mice were sacrificed using anesthetics to evaluate sperm parameters and testis histopathological alterations.

### Chemical solution preparation

Here, 15 µL DMSO was added to 1985 µL distilled water to prepare 2 ml DMSO solution to be administered to the sham group. Also, 1 mL DMSO was added to CPF powder vial (1 mg) in order to prepare CPF stoke solution (1mg/1mL). Afterward, 15 µL CPF was added to 135 µL distilled water and after pipetting, the whole solution was added to 1850 µL distilled water to prepare 2 ml High CPF (5 mg/kg.b.w) solution. Eventually, 200 µL of high CPF solution was added to 1800 µL distilled water to prepare low CPF (0.5 mg/kg.b.w) solution. NAC was dissolved in water at 35 mg/kg.b.w. It should be noted that fresh CPF solutions were daily prepared.

### Sperm motility, count and morphology assessment

Seven days after the last day of treatment, mice were anaesthetized via an inhalation induction chamber and sacrificed. Right testis of each animal was excised and put in 10% formalin solution for histopathological evaluations. Afterward, the caudal of left epididymis of each animal was excised and put in petri dish containing 3 mL Ham’s F10 (St. Louis, MO, USA) [Lot No. 87120401]. According to diffusion method (16), for assessment of sperm parameters, epididymis was tattered to smaller pieces using sterile needle syringe and kept in a CO_2_ incubator at 37°C for almost 30 minutes. Then, sperm parameters including sperm count, motility and morphology were evaluated under light microscopy.

From semen samples prepared by diffusion method, almost 50 µL semen from each mouse was smeared by a pipette on a slide. Afterwards, maximum 100 sperms were observed on right upper quarter of each slide to examine sperm count, motility and morphology. Sperm normality percentage for each mouse was easily calculated using a counter by knowing about mice sperm abnormalities (16). It was very important that well-mixed semen sample was spread at appropriate thickness on each slide to evaluate sperm parameters. During sperm assessment, room temperature was maintained at 21-24°C because increased temperature may enhance semen degeneration speed.

### Histopathological examinations

Testis specimens were kept in 10% neutral buffered formalin. For testis histopathological evaluations, 5 µm sections were prepared from each testis, stained with haematoxylin and eosin (H&E) and observed under a light microscope. Images were captured by Olympus optical microscope equipped with a Canon HD camera at magnifications ×4, ×10 and also ×40 at four random points. Afterwards, data were evaluated on a proper personal computer using Motic software instruction (17). Numbers of spermatogonia, spermatocytes and spermatid cells and also seminiferous tubules area and diameter were observed by using Motic histomorphometric utility options.

### Statistical analysis 

Data were presented as mean ± standard error (SE). Statistical analysis was performed in SPSS (version 22, SPSS Inc., Chicago, IL) using one-way analysis of variance (ANOVA) followed by Tukey as the post hoc test.

**Table 1:** Effect of NAC on sperm parameters in CPF-induced mice


	Control	DMSO	NAC	Low CPF	High CPF	Low CPF+NAC	High CPF+NAC

Sperm motility (%)	75.83 ± 0.83	71.66 ± 1.66	75.83 ± 0.83	45 ± 2.23^a^	33.33 ± 4.77^a^	75 ± 0.1^b^	57 ± 4.03^b^
Sperm normality (%)	66 ± 2.73	62.50 ± .17	63.66 ± 1.02	54 ± 1.98^a^	30.66 ± 0.7^a^	59.16 ± 1.60^b^	62.83 ± 1.85^b^
Sperm count (sperm cell concentration/ml)	87.5 ± 3.09	90 ± 4.47	93.33 ± 4.21	55 ± 2.23^a^	65.83 ± 5.23^a^	82.5 ± 3.59^b^	70 ± 2.58


The data are presented as mean ± SE (n=6). Sperm count is expressed as number×10^6^ per caudal epididymis. ^a^; Indicates a significant difference as compared to control group (P<0.05), ^b^; Indicates a significant difference as compared to CPF group (P<0.05), NAC; N-Acetyl Cysteine, CPF; Chlorpyrifos, and DMSO; Dimethyl sulfoxide,

## Results

### Morbidity and mortality

Male mice that received CPF (0.5 and 5 mg/kg.b.w/day) for 35 days showed signs of toxicity such as salivation, diarrhea and tremor. No death was recorded throughout the study period.

### Sperm characteristics

According to our data, no significant differences were found in sperm characteristics between DMSO and control group (Table 1, P>0.05). Administration of CPF 0.5 mg/kg.b.w/day (low CPF) showed significant decreases in sperm motility, count and also morphology (P<0.0001). In addition, sperm characteristics considerably decreased following administration of CPF 5 mg/kg.b.w/day as compared to control group (P<0.0001). Treatment with NAC alone made no significant changes to motility, counts and morphology. However, NAC treatment in combination with low CPF caused significant increases in motility and counts and markedly improved sperm morphology as compared to control CPF-induced groups (P<0.0001). NAC also caused significant increases in motility and improvements in morphology when co-administered with high CPF (P<0.0001), while sperm count showed no significant increases.

### Histomorphometry

**Fig.1:**
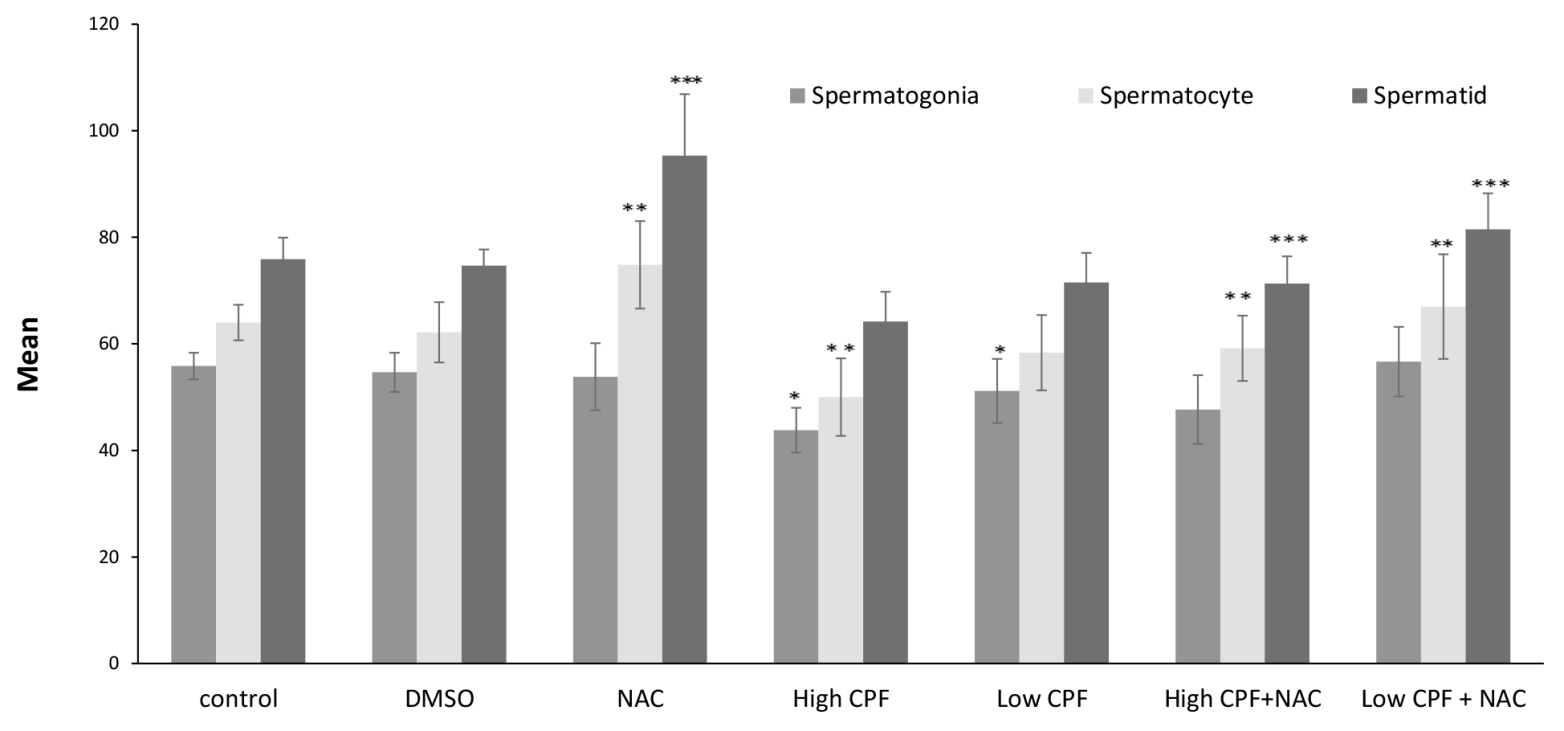
Bars presents mean ± SE of spermatogonia, spermatocytes and spermatid cells counts in different groups. *; Indicates a significant difference in spermatogonia counts as compared to control group (P<0.05), **; Indicates a significant difference in spermatocytes counts as compared to control group (P<0.05), ***; Indicates a significant difference in spermatids counts as compared to control group (P<0.05), NAC; N-Acetyl Cysteine, CPF; Chlorpyrifos, and DMSO; Dimethyl sulfoxide.

Average count of spermatogonia, spermatocytes and spermatid in DMSO group slightly decreased but it was not significant; however, a significant increase was observed in NAC group (P<0.001, Fig .1). It was demonstrated that mean number of spermatogonia cells significantly decreased in low CPF group (P<0.04). Meanwhile in High CPF group, spermatogonia, spermatocytes and spermatids were considerably decreased (P<0.0001). Treatment of CPF groups with NAC resulted in significant increases in the average number of spermatocytes and spermatids (P<0.001 and P<0.007, respectively). However, mean of spermatogonia cells counts in high CPF+NAC group had no significant increase (P<0.05).

**Fig.2:**
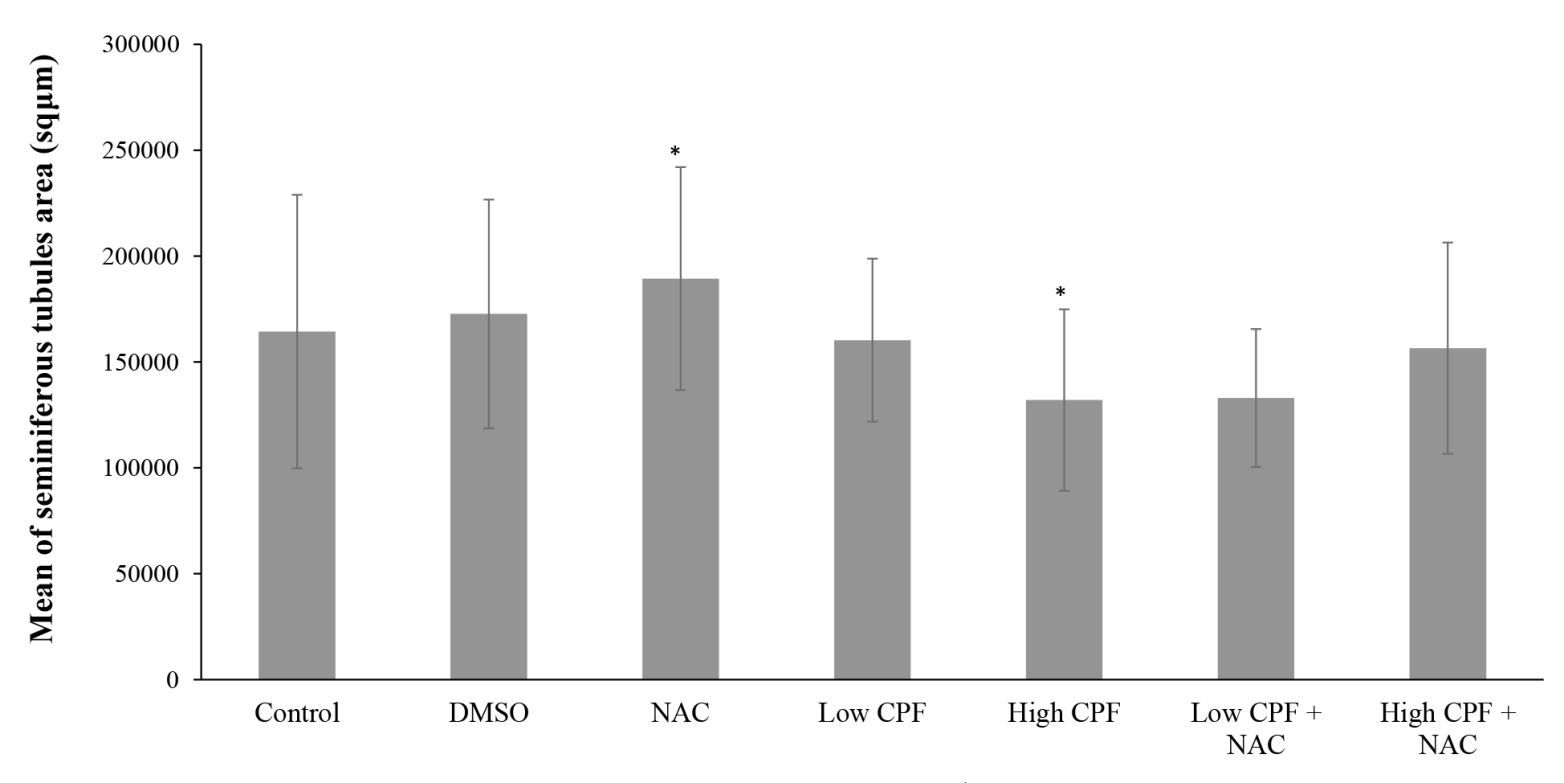
Bars presents mean ± SE of seminiferous tubules area in different groups. *; Indicates a significant difference as compared to control group (P<0.05), NAC; N-Acetyl Cysteine, CPF; Chlorpyrifos, and DMSO; Dimethyl sulfoxide,

**Fig.3:**
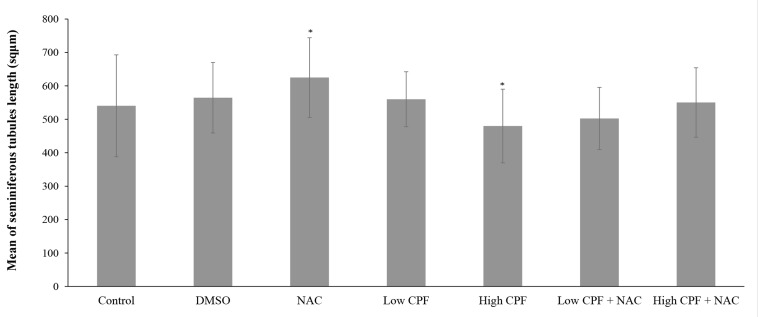
Bars presents mean ± SE of seminiferous tubules diagonal length in different groups. *; Indicates a significant difference as compared to control group (P<0.05), NAC; N-Acetyl Cysteine, CPF; Chlorpyrifos, and DMSO; Dimethyl sulfoxide.

Based on data given in Figures 2 and 3, there was no significant increase in mean seminiferous tubules area and diameter in DMSO group compared to control (P>0.05); but, NAC showed a significant increase in both variables (P<0.001). While high CPF treatment significantly diminished seminiferous tubules, low CPF treatment (0.05 mg/kg.b.w) caused no considerably damage in seminiferous tubules shape. NAC could not ameliorate the effects caused by high CPF (P>0.05).

**Fig.4:**
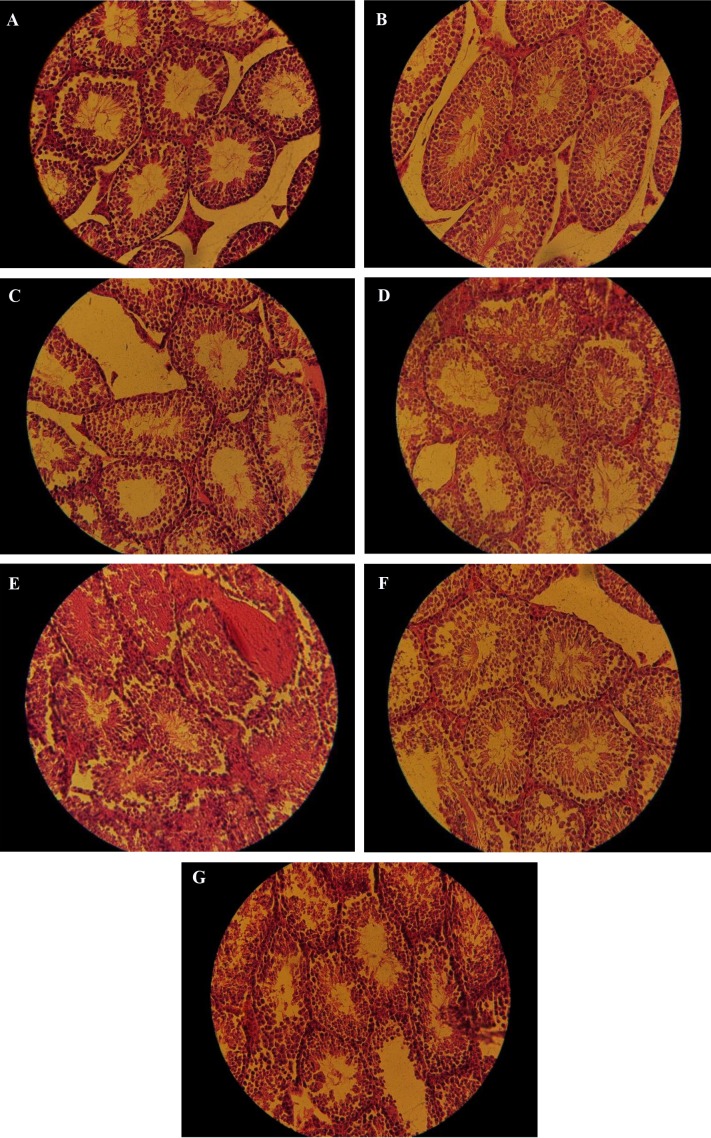
Histopathological difference is shown between experimental groups. It is demonstrated a massive destruction in CPF groups. However NAC considerably improved histopathology of testis. **A.** Control, **B.** DMSO, **C.** NAC, **D.** Low CPF, **E.** High CPF, **F.** Low CPF+NAC, and **G.** High CPF+NAC. NAC; N-Acetyl Cysteine, CPF; Chlorpyrifos, and DMSO; Dimethyl sulfoxide.

Based on data given in Figure 4, there was no significant increase in mean seminiferous tubules area and diameter in DMSO group compared to control (P>0.05); but, NAC showed a significant increase in both variables (P<0.001). While high CPF treatment significantly diminished seminiferous tubules, low CPF treatment (0.05 mg/kg.b.w) caused no considerably damage in seminiferous tubules shape. NAC could not ameliorate the effects caused by high CPF (P>0.05).

## Discussion

In CPF-exposed mice, a considerable reduction in sperm parameters was found. Meanwhile, NAC could not significantly improve sperm motility, morphology and count. NAC in combination with CPF 5 mg/kg.b.w. considerably prevented further damages to sperm motility and morphology; However, NAC could not improve sperm counts caused by CPF-induced toxicity. In a similar study on CPF reproductive toxicity, a significant decrease in sperm motility and counts was observed by CPF gavage at 20 mg/kg.b.w (18).

In addition, CPF considerably decreased the level of antioxidant enzymes and glutathione in plasma. Nigella sativa oil can act like NAC as a potent protective agent which statistically improved sperm parameters, antioxidant enzymes activity and testosterone level (18). According to our results, adverse effects of CPF is likely irreversible and has negative effects on genitalia systems.

For decades, CPF devastating effects on spermatogenesis process was unclear. Other scientists investigated various pesticides at different doses for their negative effects on spermatogenesis process (19). Organophosphates or any other chemicals, such as different toxins, that have adverse effects on tissues and cells may be inadvertently absorbed through the skin or digestion system. In order to confront these harmful effects, consumption of antioxidant substances like syrup of Malva sylvestris (20) along with hydroalcoholic extract of *Fumaria parviflora* (21) or products such as propolfenol (22) is highly recommended to protect against damages induced by toxins, particularly against those cause in the genital system. However, some antioxidant materials such as catechin and quercetin did not have significant influences in this regard (9). Nevertheless, natural nutrients such as ginger and cinnamon could be effective on male genital dysfunction due to their anti-oxidant efficacy (23).

Vitamin C and E have been widely used in previous studies and were introduced as beneficial protective materials to compensate damages induced by organophosphates such as malathion, a broad spectrum organophosphate pesticide that could decrease sperm parameters and induce histopathological alteration (24). Although vitamin C and E are potent protective materials against various toxins, a lower dose of intraperitoneal NAC possibly has a more marked impact on sperm parameters based on our findings but use of food or fruits overfilled with these vitamins is suggested for people who are daily exposed to pesticides (25). Another study showed that vitamin C only resulted in a significant improvement of sperm motility (26). 

The present study indicates that NAC at 35 mg/kg.b.w somehow significantly increased seminiferous tubules area, diagonal diameter, and spermatogonia, spermatocytes and spermatids counts. Meanwhile CPF 0.5 mg/ kg.b.w could not considerably reduce seminiferous tubules area and diagonal diameter. Furthermore, CPF 5 mg/kg.b.w significantly diminished seminiferous tubules.

Based on these findings, NAC in combination with CPF ameliorates the pesticide’s adverse effects on testis. It seems that intraperitoneal injection of NAC, even at a low dose has a more marked effect on sperm parameters than gavage administration (15). It has been proven that NAC can affect lipid peroxidation (LPO) (27). Therefore, NAC might decrease ROS elevation caused by CPF. However, in the present study, NAC exact effects on sexual hormones or anti-oxidant enzymes such as superoxide dismutase, catalase, or glutathione in treated groups, were not evaluated. But considering significant reductions in testis germinal cells, oxidative stress level was probably elevated by CPF and NAC ameliorated the adverse effect of CPF on the testis.

According to our results, seminiferous tubules area and diagonal diameters were not affected by CPF. It suggests that resting times at the end of each week and also seven days after the last injection of CPF might provide a chance for the immune system to recover and regenerate genital and possibly other tissues. Therefore we did not expect NAC to protect these two unaffected variables. Meanwhile, we assume that CPF at the dose of 0.5 mg/kg.b.w could not significantly diminish seminiferous tubules area following four-week administration. Maybe by longer treatment periods, CPF could induce more destructive effects at the dose of 0.5 mg/kg.b.w It is clear that NAC is able to confront negative effects of CPF toxicity in male genital system but what if we could use NAC at doses higher than 35 mg/kg.b.w? In this case, we probably observe NAC protective effects against CPF typical tissue toxicity. Further in vivo studies using intraperitoneal injections, are highly recommended to affirm our data. 

## Conclusion

Both low and high doses of CPF can decrease sperm parameters. Also, this pesticide at 5 mg/kg.b.w dose significantly diminishes the length and diagonal diameter of seminiferous tubules. NAC significantly improved CPF adverse effects on sperm parameters and spermatogenesis cells except spermatogonia. However, this antioxidant could not statistically ameliorate the histopathological alterations of seminiferous area induced by CPF.

## References

[1] Mascarenhas MN, Flaxman SR, Boerma T, Vanderpoel S, Stevens GA (2012). National, regional, and global trends in infertility prevalence since 1990: a systematic analysis of 277 health surveys. PLoS Med.

[2] Joshi SC, Mathur R, Gulati N (2007). Testicular toxicity of chlorpyrifos (an organophosphate pesticide) in albino rat. Toxicol Ind Health.

[3] Peiris DC, Dhanushka T (2017). Low doses of chlorpyrifos interfere with spermatogenesis of rats through reduction of sex hormones. Environ Sci Pollut Res Int.

[4] Sai L, Li X, Liu Y, Guo Q, Xie L, Yu G (2014). Effects of chlorpyrifos on reproductive toxicology of male rats. Environ Toxicol.

[5] Mavedati O, Bandariyan E, Aminashayeri S, Sergez TM, Beigi BA (2015). β-Cryptoxanthin ameliorates the reproductive toxicity of chlorpyrifos in male rabbit. Comp Clin Path.

[6] Ojha A, Srivastava N (2014). In vitro studies on organophosphate pesticides induced oxidative DNA damage in rat lymphocytes. Mutat Res Genet Toxicol Environ Mutagen.

[7] Li D, Huang Q, Lu M, Zhang L, Yang Z, Zong M (2015). The organophosphate insecticide chlorpyrifos confers its genotoxic effects by inducing DNA damage and cell apoptosis. Chemosphere.

[8] Chebab S, Mekircha F, Leghouchi E (2017). Potential protective effect of Pistacia lentiscus oil against chlorpyrifos-induced hormonal changes and oxidative damage in ovaries and thyroid of female rats. Biomed Pharmacother.

[9] Uzun FG, Demir F, Kalender S, Bas H, Kalender Y (2010). Protective effect of catechin and quercetin on chlorpyrifos-induced lung toxicity in male rats. Food Chem Toxicol.

[10] Altun S, Özdemir S, Arslan H (2017). Histopathological effects, responses of oxidative stress, inflammation, apoptosis biomarkers and alteration of gene expressions related to apoptosis, oxidative stress, and reproductive system in chlorpyrifos-exposed common carp (Cyprinus carpio L.). Environ Pollut.

[11] Feng D, Huang H, Yang Y, Yan T, Jin Y, Cheng X (2015). Ameliorative effects of N-acetylcysteine on fluoride-induced oxidative stress and DNA damage in male rats’ testis. Mutat Res Genet Toxicol Environ Mutagen.

[12] Duarte F, Blaya R, Telöken PE, Becker D, Fernandes M, Rhoden EL (2010). The effects of N-acetylcysteine on spermatogenesis and degree of testicular germ cell apoptosis in an experimental model of varicocele in rats. Int Urol Nephrol.

[13] El-Maddawy ZK, El-Sayed YS (2018). Comparative analysis of the protective effects of curcumin and N-acetyl cysteine against paracetamol-induced hepatic, renal, and testicular toxicity in Wistar rats. Environ Sci Pollut Res Int.

[14] da Silva RF, Borges Cdos S, Villela E Silva P, Missassi G, Kiguti LR, Pupo AS (2016). The coadministration of N-acetylcysteine ameliorates the effects of arsenic trioxide on the male mouse genital system. Oxid Med Cell Longev.

[15] Farombi EO, Ugwuezunmba MC, Ezenwadu TT, Oyeyemi MO, Ekor M (2008). Tetracycline-induced reproductive toxicity in male rats: effects of vitamin C and N-acetylcysteine. Exp Toxicol Pathol.

[16] Seed J, Chapin RE, Clegg ED, Dostal LA, Foote RH, Hurtt ME (1996). Methods for assessing sperm motility, morphology, and counts in the rat, rabbit, and dog: a consensus report.ILSI risk science institute expert working group on sperm evaluation. Reprod Toxicol.

[17] Kazemi S, Feizi F, Aghapour F, Joorsaraee GA, Moghadamnia AA (2016). Histopathology and histomorphometric investigation of bisphenol A and nonylphenol on the male rat reproductive system. N Am J Med Sci.

[18] Mosbah R, Yousef MI, Maranghi F, Mantovani A (2016). Protective role of Nigella sativa oil against reproductive toxicity, hormonal alterations, and oxidative damage induced by chlorpyrifos in male rats. Toxicol Ind Health.

[19] MahdinezhadGorji N, Jorsaraei SGA, Hojati V, Zabihi E, Khalilpour A, Abedian Z (2015). A comparison between the cytotoxicity induced by gossypol in two testicular cell lines. Iranian Journal of Toxicology.

[20] Saad AB, Rjeibi I, Alimi H, Ncib S, Smida A, Zouari N (2017). Lithium induced, oxidative stress and related damages in testes and heart in male rats: the protective effects of malva sylvestris extract. Biomed Pharmacother.

[21] Shokoohi M, Shoorei H, Soltani M, Abtahi-Eivari SH, Salimnejad R, Moghimian M (2018). Protective effects of the hydroalcoholic extract of Fumaria parviflora on testicular injury induced by torsion/detorsion in adult rats. Andrologia.

[22] Biagi M, Collodel G, Corsini M, Pascarelli NA, Moretti E (2018). Protective effect of Propolfenol® on induced oxidative stress in human spermatozoa. Andrologia.

[23] Khaki A, Khaki AA, Hajhosseini L, Golzar FS, Ainehchi N (2014). The anti-oxidant effects of ginger and cinnamon on spermatogenesis dys-function of diabetes rats. Afr J Tradit Complement Altern Med.

[24] Uzun FG, Kalender S, Durak D, Demir F, Kalender Y (2009). Malathion-induced testicular toxicity in male rats and the protective effect of vitamins C and E. Food Chem Toxicol.

[25] Khaki A, Ghanbari Z, Ghanbari M, Ouladsahebmadarek E, Javadi L, Farzadi L (2011). Anti-oxidative effects of citro flavonoids on spermatogenesis in rat. Afr J Pharm Pharmacol.

[26] Dirican EK, Kalender Y (2012). Dichlorvos-induced testicular toxicity in male rats and the protective role of vitamins C and E. Exp Toxicol Pathol.

[27] Yang R, Le G, Li A, Zheng J, Shi Y (2006). Effect of antioxidant capacity on blood lipid metabolism and lipoprotein lipase activity of rats fed a high-fat diet. Nutrition.

